# PCCNoC: Packet Connected Circuit as Network on Chip for High Throughput and Low Latency SoCs

**DOI:** 10.3390/mi14030501

**Published:** 2023-02-21

**Authors:** Xinbing Zhou, Peng Hao, Dake Liu

**Affiliations:** 1 School of Information and Communication Engineering, Hainan University, Haikou 570228, China; 2 School of Computer Science, Northwestern Polytechnical University, Xi’an 710072, China; 3 Ultichip Communications Technology Company Limited, Beijing 100191, China

**Keywords:** Network on Chip (NoC), packet connected circuit, low latency

## Abstract

Hundreds of processor cores or modules are integrated into a single chip. The traditional bus or crossbar is challenged by bandwidth, scalability, and silicon area, and cannot meet the requirements of high end applications. Network-on-chip (NoC) has become a very promising interconnection structure because of its good scalability, predictable interconnect length and delay, high bandwidth, and reusability. However, the most available packet routing NoC may not be the perfect solution for high-end heterogeneous multi-core real-time systems-on-chip (SoC) because of the excessive latency and cache cost overhead. Moreover, circuit switching is limited by the scale, connectivity flexibility, and excessive overhead of fully connected systems. To solve the above problems and to meet the need for low latency, high throughput, and flexibility, this paper proposes PCCNoC (Packet Connected Circuit NoC), a low-latency and low-overhead NoC based on both packet switching (setting-up circuit) and circuit switching (data transmission on circuit), which offers flexible routing and zero overhead of data transmission latency, making it suitable for high-end heterogeneous multi-core real-time SoC at various system scales. Compared with typically available packet switched NoC, our PCCoC sees 242% improved performance and 97% latency reduction while keeping the silicon cost relatively low.

## 1. Introduction

It is expected that CMOS will be the mainstream digital integrated circuit technology within the next 20 years. The clock frequency of CMOS digital systems tends to remain at a reasonable rate instead of significantly improving due to the saturation of Moore’s Law [1]. Thus, parallel architecture and parallel computing are introduced to improve system performance The homogeneous parallel system on chip (SoC) can provide sufficient flexibility and performance for general applications, but its ability is inadequate for some specific domains such as high-speed real-time embedded systems (e.g., wireless communication baseband processor). Heterogeneous multi-core architectures have been introduced to improve performance in dedicated domains, and have gradually become the hotspot of the market [2]. The characteristics of heterogeneous multi-core real-time SoC are as follows: (1) specially designed for stable application software in a specific application domain; (2) ultra-high computing performance requirements.

Heterogeneous multi-core real-time SoC needs a characteristic network on chip that can provide relatively predictable and stable data flow. The heterogeneous multi-core real-time SoC needs a particular on-chip connection method which could provide a relatively predictable and stable data flow. the connection method shall at least meet the following requirements: (1) extremely wide bus width with hundreds of bits; (2) concurrent multiple data flow transmission; (3) ultra-short setup time; (4) stable and orderly data transmission; (5) low overhead non-disordered transmission without cache; (6) negligibly low power consumption compared with the power consumption of the computing and storage; (7) flexible and dynamic connection capability; (8) enough nodes and ports to provide full connection; (9) scalable and reconfigurable distributed connections; (10) a reliable and deadlock-free network.

**Figure 1:**
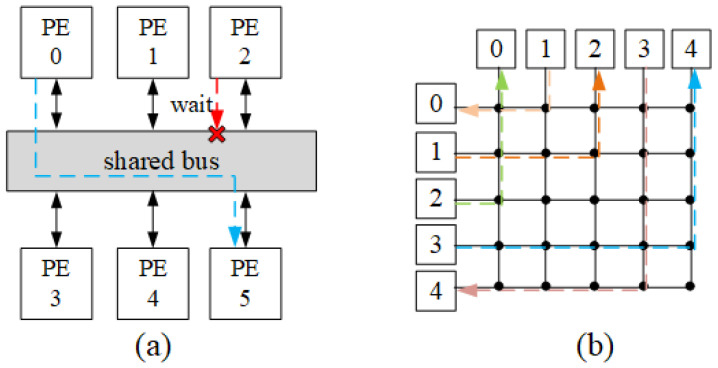
Architecture for (**a**) shared bus and (**b**) fully-connected crossbar. PE means processing element. (**a**) shows that PE2 with low priority has to wait when the bus is occupied, while (**b**) shows the fully connection of all PEs.

When the number of processor cores is relatively small, bus and crossbar are traditional interconnection methods [3]. All processing elements (PEs) share the transmission medium when using shared bus as shown in Figure 1a. When the number of PE connected to the bus increases, the bus traffic will quickly reach the saturation state, and the system is difficult to obtain higher bandwidth, which cause the poor scalability of the bus. The shared bus has relatively low transmission efficiency because it doesn’t support parallel data transmission, but it requires relatively low power consumption. Crossbar provides fully connections between all PEs with multiplexers as depicted in Figure 1b. Although crossbar can solve the transmission parallelism problem, its scalability is poor due to the area overhead and power consumption with the increase of the PE number.

As more and more cores are integrated on a single chip, traditional interconnect architectures hit the bottleneck, and the NoC interconnection architecture emerges [4,5]. The NoC architecture perfectly solves the defects of traditional interconnect schemes. The advantages of NoC are mainly reflected in the following three aspects. First, NoCs provide enough concurrency for communication between PEs on chip. Second, compared with the bus and crossbar architecture, the NoC architecture has strong scalability, and can easily expand the topology distribution according to the rules of the original topology. Third, the latency of NoC communication is low.

The existing NoC architectures are mainly divided into two categories: packet switching (PS) NoC and circuit switching (CS) NoC. PS NoC transmits data packets through dynamic routing, and the receiver of PS NoC may have to reorder the packets. CS NoC transmits and receives data by establishing a circuit-based data path [6].

The characteristics of existed PS NoCs are as follows: (1) the data transmitted at one time is divided into several small flits (Flow control unIT), and each flit carries the active status, destination addresses and data information (such as the order and length of the packets). (2) Reordering and error-checking are required at the receiving end. (3) Small packet transmission based on short data has better latency. PS NoC has brought many problems too, including: (1) Packet switching itself cannot guarantee the order of arriving packets, so that the receiver should be equipped with a reorder buffer, which greatly increases the hardware overhead and transmission delay; (2) Due to the limitation of hardware and reliability, the data packet cannot be too long, so that the transmission proportion of the header is relatively large and the transmission efficiency is low; (3) The transmission time is long, redundant and uncertain because each packet must establish its own route, which is not suitable for real-time systems, which make PS NoCs are not suitable for real-time systems [5,6].

The advantages of CS NoCs are that the transmission time is low after the circuit eatablished, and there is no need to reorder the cache. But CS NoC is master-controlled and is only suitable for small-scale systems with few connections. The connection ability of CS NoC depends on the chip circuit design, which leads to little design margin for later stage design. Additionally, the circuit setup and release time are large, so the process redundancy of short data packets are large [6].

The above-mentioned PS NoCs and CS NoCs both have limitations for heterogeneous multi-core real-time SoC. Therefore, this paper proposes a novel NoC architecture that combines the routing capability feature of PS NoC and low transmission latency character of CS NoC, which is suitable for heterogeneous multi-core real-time SoC.

The rest of the paper is organized as follows. Section 2 briefly describes some PS NoCs, CS NoCs and previously proposed hybrid switching mechanisms. Section 3 introduces the proposed architecture. Section 4 discusses the workflow of the proposed mechanism. Section 5 discusses simulation parameters and results of the proposed router architecture and the synthesis result. Finally, Section 6 draws the conclusion.

## 2. Related Work

**Figure 2:**
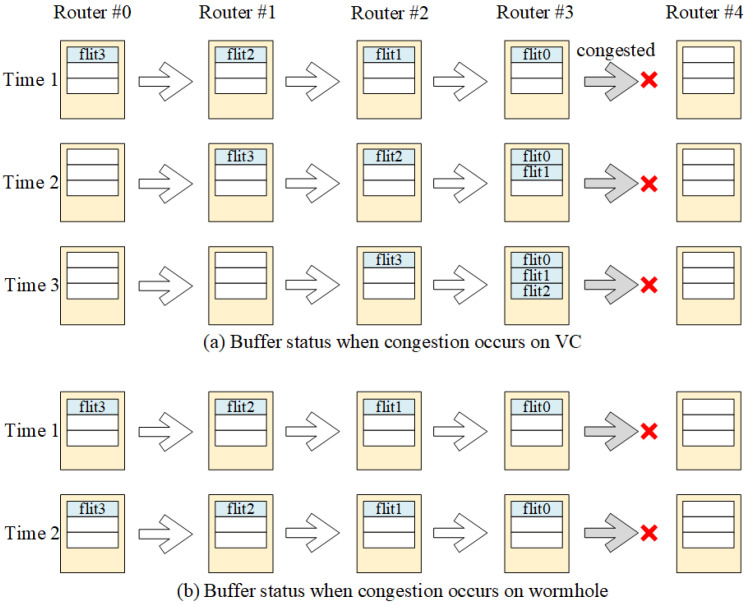
The flit status when the is network congested for (**a**) virtual channel PS NoCs and (**b**) wormhole PS NoCs.

There are many packet-switched NoCs and Circuit-switched NoCs in the literature. PS NoC has been intensively studied by researchers, and there are several kinds of PS NoC [7,8,9,10,11,12,13,14,15], mainly including virtual channel (VC) and wormhole (WH) switching. In the virtual channel technique, a packet is decomposed into flits, which are then routed consecutively through the network. The adjacent router needs to store the whole packet when network congested in VC as described in Figure 2a. The memory size takes up a large area of the system on chip. The wormhole switching technique reduces the usage of the buffer, as it uses backpressure to stall the route path when the network congestion happens as shown in Figure 2b. However, segmented storage of flits is more likely to cause link blocking and deadlock [16].

Generally speaking, wormhole switching NoCs with virtual channels is well accepted and utilized [10]. Dally’s book [5] has covered almost every aspect of such a PS NoC, while Dake’s book [17] has mentioned the design methodology of such NoCs.

VCS [7] (virtual circuit switch) uses a flow-control policy that exclusively reserves a virtual path for routing packets in an adaptive mode for each communication flow in order to guarantee that delivery of all packets belongs to the same communication flow. It allows sharing of the reserved path among packets of different communication flows. The method needs additional memory unit for storing all unordered packets, and needs extra channels to for path reservation.

MRBS [9] manages all input buffers in a router as a register file style instead of dedicating a single buffer per input port, which efficiently utilizes the buffer space, especially for multicast traffic patterns. MRCN [8] uses destination router partitioning and traffic-aware adaptive branching technique to reduce packet routing hops and disperse channel traffic. It can ensure deadlock freedom by utilizing extended routing and router labeling rules. DancerFly [15] is an order-aware network-on-chip router which resolves out-of-order packet delivery issue in two steps. First, it performs in-buffer reordering by reordering packets queuing in the input buffer. Second, packets from different input ports are reordered before going through the router. Ref. [18] proposes a solution that guarantees in-order packet delivery while packets are routed through multiple paths in the network, and [19] proposes a solution that supports in-order packet delivery under adaptive routing by reserving an alternate virtual path during the VC allocation process. Yet, such approaches are relatively expensive to implement in terms of silicon footprint and have high power consumption due to the extensive use of buffers.

There are studies in the literature studying adaptive NoCs [19,20] to improve application parallelism abilities and fast path NoCs [21,22,23], which improves latency and throughput by redesigning the PS routers. Adapt-NoC [20] introduces a reinforcement learning (RL)-based control policy. It can dynamically allocate several disjoint regions of the NoC, called subNoCs, which can provide efficient communication support for concurrent application execution. Adapting NoC can handle a variety of traffic patterns for running multiple applications at the same time. It is capable of adapting to a given topology such as a mesh, cmesh, torus, or tree, thereby tailoring the topology [19]. The above-mentioned studies represent a hot research domain. Nevertheless, there are drawbacks in terms of uncertain transmission times for real-time applications.

Multi-hop bypassing has been researched intensively to design a near-optimal interconnection network [21]. FastPass [22] promotes a packet to traverse the network bufferlessly, and provides multiple pre-defined non-overlapping lanes. FastPass can transfer packets via the pre-defined non-overlapping lanes to guarantee that the packet is not blocked by congestion or deadlock. Extra lanes are need for routers, which increases the design complexity of SoC. FastTrackNoC [23] presents a method of bypassing the VC switch traversal (ST) stage to reduce packet latency. It adds a fast-track path to a 2D mesh router between the head of a single input virtual channel (VC) buffer and its most used opposite output to reduce router processing time.

CS NoCs mainly use the fully customized network connections method [24,25,26,27,28,29]. Fully customized network connections are based on static communication task graphs. However, as the flexibility of such architectures is limited to a specific application, they lack flexibility in modern many-core architectures, where multiple tasks of applications are scheduled to run concurrently. As a result, they may not support applications such as H.264 [30] which requirement dynamic communication setups.

Many hybrid NoCs have been proposed in recent research. The connection setup of hybrid switch networks can be implemented by a packet configuration [31,32,33,34] or by a master-controlled dedicated network configuration [35,36,37,38].

In [32], a technique called Hybrid Circuit Switching “HCS” is presented. It consists of a network setup which handles the construction and reconfiguration of circuits and then stores the switch configuration for active circuits, along with a data network for packet transferring which intermingles circuit-switched flits with packet-switched flits. The packet does not wait for an acknowledgment that a circuit has been successfully constructed. If there are no unused circuit planes, incoming circuit-switched (CS) flits are tagged as packet-switched flits and remain packet-switched until their destination. QoS cannot be guaranteed, as the router can change the packet type dynamically.

In [34], a TDM (time division multiplexing) circuit-switched part for transferring Guaranteed Service (GS) packets and a packet-switched part for transferring best effort (BE) packets were proposed. The packet switched network utilizes the unreserved resources to increase resource utilization. The problem is that the setup latency is not guaranteed, because of the unpredictable contention of best-effort packets.

In the CirKet switching mechanism [35], messages are divided into two different priority classes: high priority (HP) messages and low priority (LP) messages. HP packets have a higher priority as compared to LP packets in obtaining network resources. HP uses pseudo-circuit switching that does not share network resources with other communications. LP changes crossbar configuration to establish new link communication. This requires external router calculation time, as LP and HP can be transferred simultaneously.

PCSAT_NET [37] is composed of a configuration network (Cfg_net), a status network (Stat_net), and a data network (Data_net). It uses a dedicated configuration network for communication setup and a specific state network for state information transferring. It is able to solve the problem of long path locking time, however, the trade-off in terms of increased silicon cost and time certainty has to be considered.

## 3. Proposed Architecture

As mentioned above, our NoC targeted at heterogeneous multi-core real-time SOC. The requirement of the NoC is to support relatively fixed task flows which usually have a large amount of data to transfer, require excellent real-time performance, support parallel periodic jobs, and call for short setup time.

For example, the baseband processor for wireless communication is one of the target applications, whose main tasks are the symbol domain task and bit domain task. In a certain period of time, there are multiple parallel task flows to deal with which have large data volume. Task flow changes over time, and task switching needs to be completed quickly. However, the traditional CS NoC has difficulty meeting these requirements due to the long establishment time, and the PS NoC cannot guarantee data throughput and communication quality.

We redesigned the router and network interface of traditional PS NoC to combine the merits of both circuit switching and packet switching. The router design is described in Section 3.2. The proposed packet connected circuit (PCC) as network on chip offers short setup time, flexible routing, certain transmission time, and zero overhead of data transmission latency with low area and low power overhead, which is suitable for heterogeneous multi-core real-time SoCs.

### 3.1. PCCNoC Design

**Figure 3:**
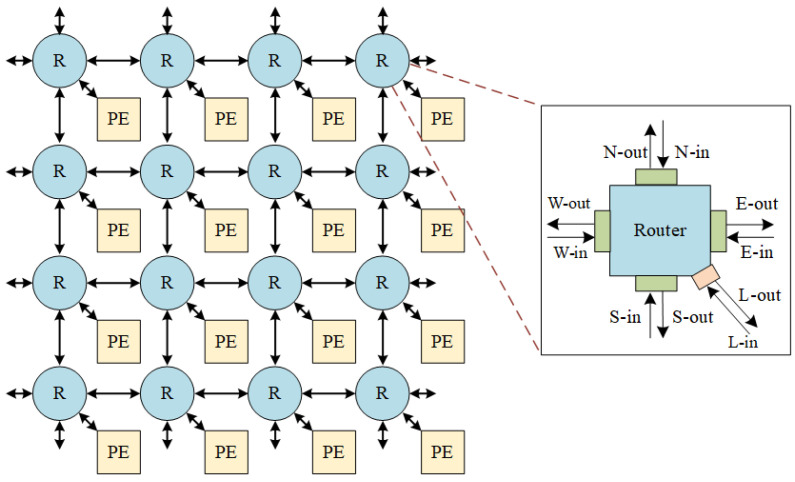
A 4 × 4 mesh NoC architecture. R represents router and PE means processing element.

The PCCNoC architecture is based on a two-dimensional mesh topology. It is composed by routers, network interfaces, and PEs. Figure 3 shows a top-level diagram of the PCCNoC with a 4 × 4 mesh structure. The router and network interface of PCCNoC are redesigned to support the brand new transmission mechanism.

A router has two functions: (1) to set up transmission circuit by routing under the new routing protocol, and (2) to use the set-up circuit for data transmission.

Inputs and outputs of each network router include five directions: East—Ein, South—Sin, West—Win, North—Nin, and a local module—Lin; East—Eout, South—Sout, West—Wout, North—Nout, and a local module—Lout, as shown in Figure 3.

The PE in heterogeneous multi-core SoC can be:

1. **Processor**. The writing bus of data storage (Lin) or program memory or configuration memory (Lin) are connected; the reading bus of data storage (Lout) is connected.
2. **Function module**. The writing bus of data storage (Lin) and configuration register (Lin) are connected; the reading bus of data storage (Lout) is connected.
3. **Memory**. The write port input to the data memory (Lin) is connected to the router; the read port of the data memory (Lout) is connected to the router.
4. **Interface (I/O)**. The router connects the interface (I/O), and the DMA inside the node provides subsequent data transmission.

### 3.2. Router Design

The main module in the NoC is the router, which is called the node. Compared with traditional NoC routers, the proposed router does not have income and outcome buffers, and it introduces a new structure called the data-path locker. When PE-A transfers data to PE-B the first time, the data-path locker locks the routing path to construct a dedicated data path. The following data transmission from PE-A to PE-B uses a dedicated data-path, similar to traditional CS NoC, to ensure lower latency. When an exception happens (e.g., time violation, CRC failed) or data transmission finishes, the established path can be quickly released and the locked path can be used for packet switching again, which makes for high flexibility similar to PS NoC. The router is re-constructed to adapt the feature of both PS NoCs and CS NoCs. The router in this design has two functions: (1) establish transmission circuit by packet routing and (2) lock the routed path to support fast circuit switching. Every node can be stated in two aspects: (1) as routing source or routing destination and (2) as a data receiver or data sender. These two aspects are uncoupled. The following abbreviations will be used afterwards.

1. **Routing source (RS)**: the node that initiates the transmission request;
2. **Routing target (RT)**: the node that accepts the transmission request;
3. **Data sender (DS)**: the node that sends data, which can be RS or RT;
4. **Data receiver (DR)**: the node that receives data, which can be RS or RT.

**Figure 4:**
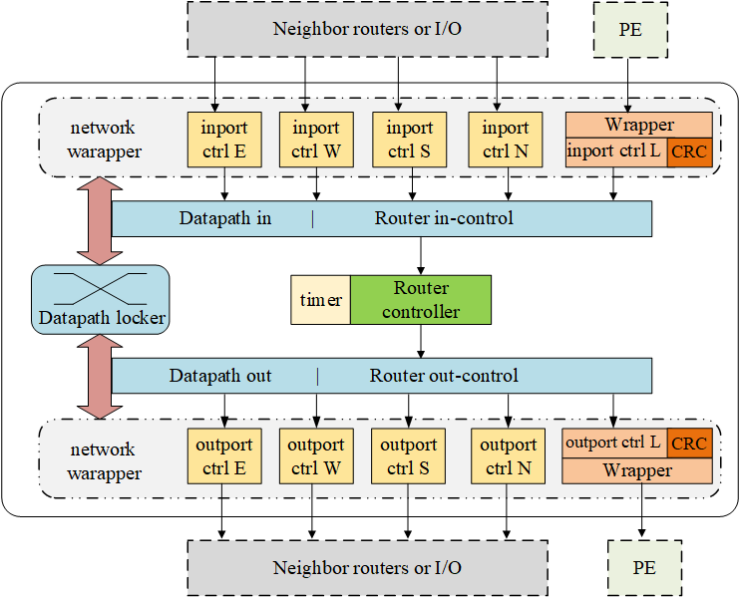
Router architecture.

The router is composed of network wrapper, router controller, timer watchdog, CRC, and datapath locker as shown in Figure 4.

**Network wrapper (NW)**: It includes module entrance configuration circuit and output configuration circuit for linking a PE to a router and connecting neighbor routers. The main modules are wrappers and port controllers for input and output which are responsible for data transmission management. To establish a new transmission, the PE sends circuit setup request to wrapper. The in-port controller would assemble a packet according to the request and start to send to the target node. If the node is data sender, before the dedicate circuit is connected, the wrapper will backpressure the local module to hold the data. After receiving the acknowledgment that the circuit has been constructed, the in-port controller will package the rest data as circuit data and sent it out through the locked datapath. If the node is data receiver, the out-port controller will unpack the packet, check the validity of the payload, and send the in-order data to local module.

**Router controller**: It is responsible for routing information processing and maintaining. If the link has been established, it will act as a transfer logic to send the data packet through the datapath locker. If there is no available constructed link, the router will parse the packet and send it to the corresponding outport or PE according to the packet information and the occupancy status of adjacent nodes. Router controller also records and keeps updating the occupancy status of adjacent nodes. If the message is configured with a request for unlinking, the router controller will send request to datapath locker to release the lock between input and output after processing the packet. Multicasting and broadcasting are also processed by router controller.

**Timer**: Before sending out the routing packet, the routing source shall reserve margin according to the routing time cost and data transmission time cost, set the timeout counter and start the countdown. When time violation happens, the routing source node will give a time violation signal to data sender and handle this failure.

**CRC** (optional): When CRC error checking is required, the CRC module at data sender completes CRC coding according to the protocol. The CRC module at the data receiver completes CRC decoding and error checking according to the protocol. If there is an error, the data receiver will report CRC error to data sender and data sender handle the failure. CRC is optional in the proposed design. The data transmission is strictly in-order after the datapath locked just as CS NoCs, so the CRC module is optional.

**Figure 5:**
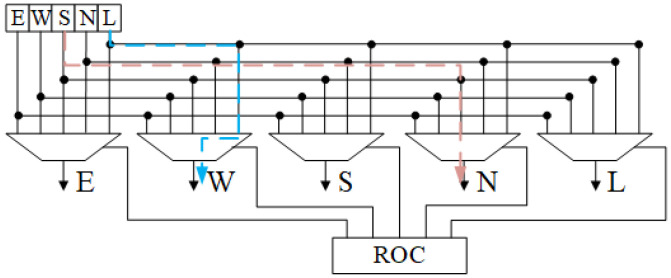
Schematic for datapath locker. The connected example shows that local-inport is connected to west outport and south inport is connected to north outport.

**Datapath locker**: It is composed of the router in-control (ROI), router out-control (ROC), and datapath locker. The routing decision is sent to the crossbar switch by the router controller to implement output selection and the relative paths are locked after sending routing packets, which become parts of the circuits to be established. The circuit structure diagram of the module is shown in the Figure 5.

### 3.3. Interface Structure

The data port of the PCCNoC interfaces can transfer two types of data. The first type is the control packet. There are four kinds of control packet: (1) route packet which is used to establish and lock routing path, (2) broadcast packet which used for global reconfiguration, (3) multicast pacekt, (4) short packet for small data transmission. The control packet transmission mechanism is similar with the conventional packet switching network, which routes the packet to the routing target by node. The second type is payload, which packs all messages to be transmitted and transmits them quickly on the locked path through circuit switching. The two types of data are transmitted use the same physical resource. If the link is locked, the data is directly forwarded by the circuit. If it is not locked, the router controller will parse the data according to its contents.

**Figure 6:**
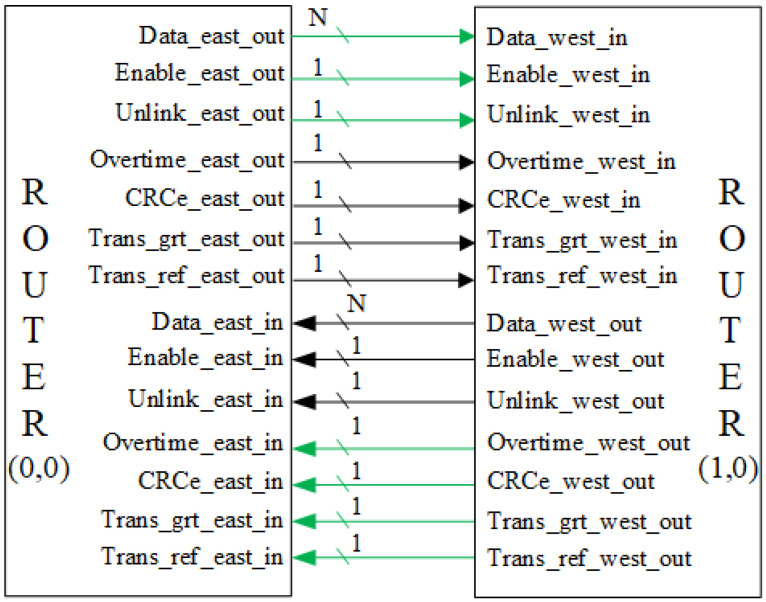
The port connection when router (0,0) establish a path with router (1,0). The green wires belong to a group and the black wires belong to another group. The green wires are the connected path. The bit width of wires are labeled on wire. The interface structure of the other three directions are the same.

The interface is composed by four groups of ports. Each group contains a bandwidth configurable (N is configurable) data port and seven control ports. The control ports are: enable (EN), unlink (UL), overtime (OT), CRC error (CRC), transmission grant (TG), transmission refuse (TR) and broadcast indicator (BC). The detailed information for each part of interface are listed in Table 1. When constructing transmission circuit, the control bits belong to the same group are connected in different directions as shown in Figure 6.

**Figure 7:**
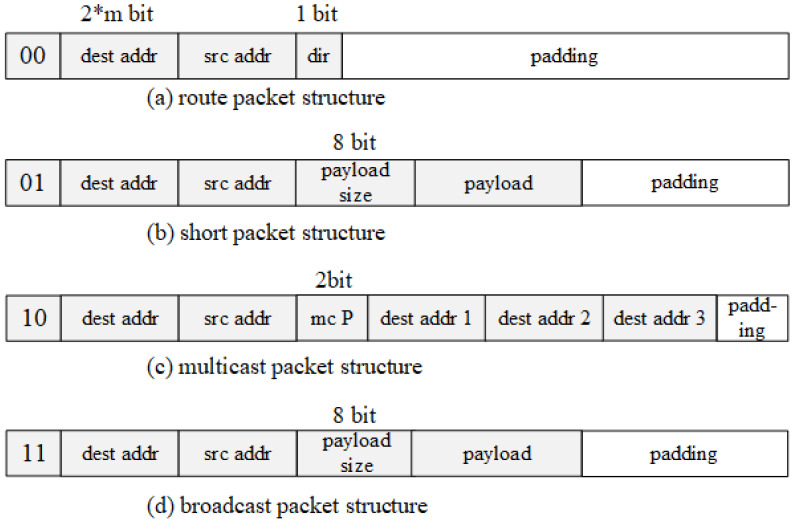
The structure of the routing packets. (**a**) route packet structure, (**b**) short packet structure, (**c**) multicast packet structure and (**d**) broadcast packet structure.

As shown in Figure 7, there are four different types of packets distinguished by the 2 bits at header: (1) a route packet, which is used to establish a transmission path; (2) a short packet, which is used for short message transmission by packet switching without constructing transmission path on the way; (3) multicast packet which is used to constructed multiple transmission circuits; (4) broadcast packet that is used to transmit broadcast information. The “m” is related to NoC size “M”. “M” is maximum between mesh row nubmer and mesh column number.

(1) $$m={log}_{2}/M$$

In this design, the router could quickly transmit control information merely by increasing 7 bits of interface bit width, instead of adding a separate set of interfaces [31,32]. The design of this work not only increases the flexibility of the interface, it reduces the complexity of the router design.

### 3.4. Routing Algorithm

Many researchers have explored routing algorithms to improve NOC performance [2,16,39]. The PCCNoC does not need to establish and revoke circuits frequently; thus, the routing algorithm is not the focus of this paper. The routing algorithm adopted by PCCNoC needs to ensure that no deadlock or livelock [16] occurs and to improve the packet exchange efficiency as much as possible. The adaptive XY routing we use is based on the traditional XY [2] routing method and has been improved.

Each network node has a unique address. The address is encoded in two segments in the east–west direction and north–south direction. The routing is guided by the the control packet. The routing algorithm adopted consists of the following steps.

1. The address codes of the two sections are X for east–west (the east is bigger) and Y for south–north (the north is bigger).
2. When routing, the address of target node is compared with the local node address. When the X address of target node is bigger, they move eastward, and vice versa; when the Y address of target node is bigger, they move northward, and vice versa;
3. The routing decision of each routing node needs to take the congestion of the surrounding routing nodes into account. The X direction has higher priority.

**Figure 8:**
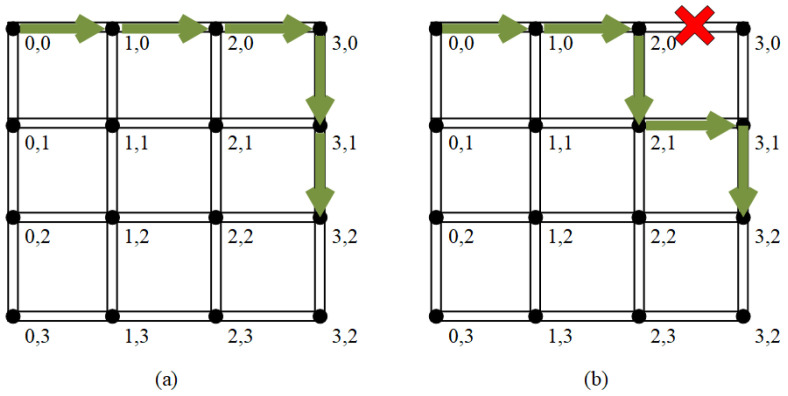
(**a**) shows the route path from (0,0) to (3,2); (**b**) shows the route path from (0,0) to (3,2) when the path from (2,0) to (3,0) is congested.

Figure 8 shows the strategy when the router faces congestion.

## 4. The NoC Workflow

The transmission mechanism of PCCNoC is specially designed to combine the advantages of both PS NoCs and CS NoCs. In this section, the four kinds of control packets shown in Figure 6 are introduced and the details of pipeline are discussed.

### 4.1. Transmission Mechanism

**Figure 9:**
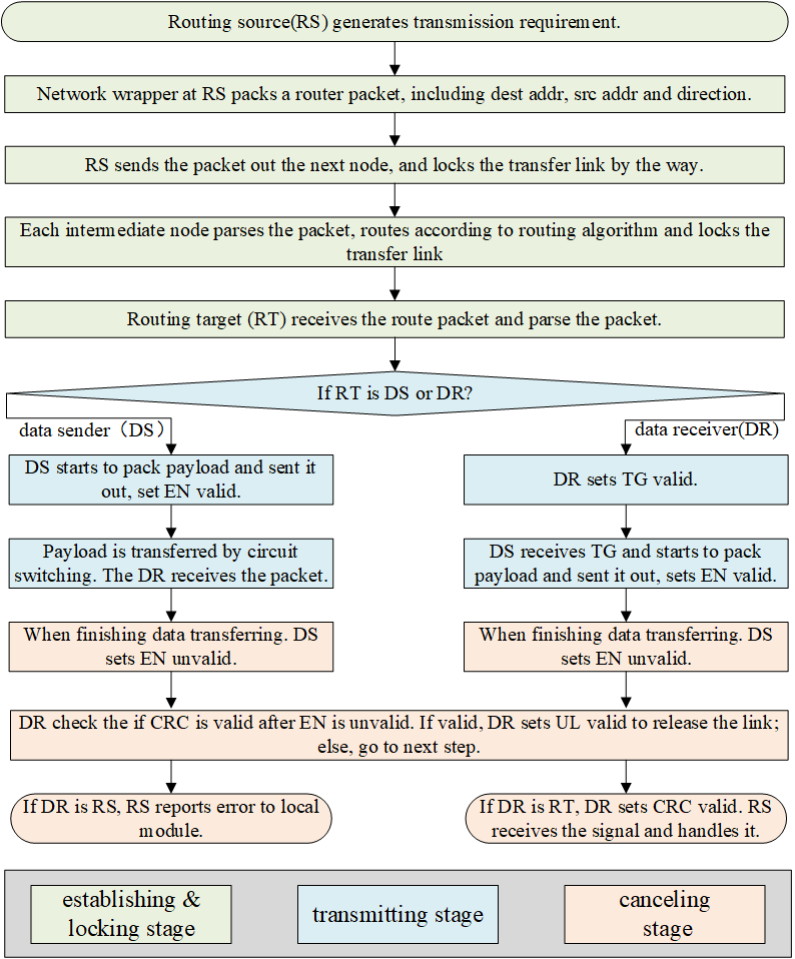
PCCNoC data transmission workflow.

The transmission task of NoC proposed in this paper contains three steps: establishing & locking, transmitting and canceling as depicted in Figure 9.

**Establishing & Locking:** The network wrapper module completes the preparation of source routing packets according to the requirements of the source routing initiate module. To establish a data transmission, the tasks for each router are as follows.

1. The router keeps a record of the occupancy of adjacent nodes.
2. According to the destination address of the route and the occupancy status of adjacent nodes, temporarily lock the inport and outport paths of the node according to data transfer direction, and send routing packets to the determined direction.
3. The routers along the transmitting path lock circuit to establish a directional locked data path.
4. The routing target node confirms the transmission request.
5. The routers along the transmitting path keep to support payload transmission.
6. The routing source node is equipped with timeout control, and the transmission with abnormal timeout will be terminated and reported to the system.

**Transmitting:** After the circuit is locked, the data sender starts to transmit payload on the circuit, and the data sender could be routing source node or routing target node.

**Canceling:** When the transmission finishes, the network wrapper module of the data sender sets enable bit unvalid. The data receiver catches the signal and sets unlink bit valid to unlock the circuit, and all nodes along the constructed path unlock and release the circuit resources.

Any nodes in the NoC can be used as a routing source. The routing source node starts and establishes a dynamic route, then temporarily locks a data transmission circuit connecting the source node to the target node. If the routing source is data sender, it starts to send data immediately when it receives the transmission grant from the data receiver. If the routing source node is a data receiver, it will receive payload and check CRC validation (if configured) and unlock the transmission path after data transmission. Every routing source is configured with a timer watchdog. If the transmission time runs out, the routing source will unlock the locket data path immediately and broadcast the timeout exception with special bits.

The intermediate node participates in data transmission, and its related routing and data transmission are completed by router controller. The router controller at each routing node (including the routing source node and the routing target node) sends the node usage status to the adjacent nodes.

Any node can be used as the routing target. The routing target node receives the route packet and starts the endpoint protocol immediately. If the routing target is data receiver, when the request of the routing source node is acceptable, the routing target returns the transmission grant signal to routing source immediately. If the request of the routing source cannot be accepted since the target node is busy, the target node immediately sends transmission refuse signal back to routing source node. If routing target is data sender, it will start to pack payload and send it out on the locked path.

### 4.2. Multicast and Broadcast

In the usage scenarios of heterogeneous multi-core SoC, such as wireless base stations, there is usually a demand for data multicasting and broadcasting. PCCNoC has multicasting function and broadcasting function.

**Figure 10:**
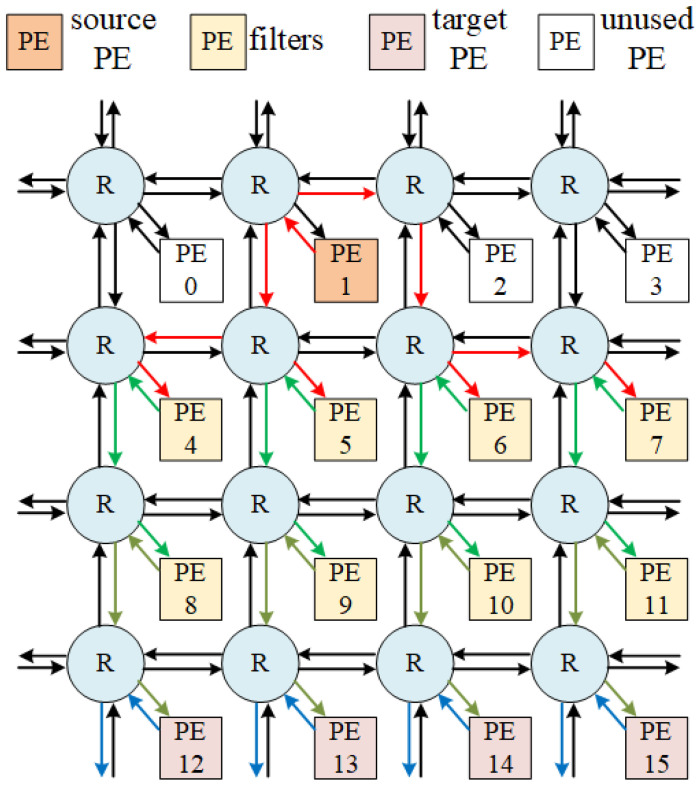
A DFE SoC with proposed NOC architecture using multicasting. The source PE generates data and performs data pre-processing. Target PEs perform data post-processing. The filters are ASIC modules. The application mapped in this diagram is a four-antenna transmission circuit for 60 MHz bandwidth. The direction of the arrows represents the data flow direction. PE3∼PE7 receive multicasting data from PE1.

The multicast packet structure is specified in Figure 7c. When using the multicasting function, the multicasting number P is set during the route establishment period. During the routing process, every time a routing target is reached, it sends a transmission grant (TG) to the routing source. The routing source node starts data transmission after receiving P TG signals. After the routing source node finishes sending data, it sends an unlink signal to all routing targets. For multicasting and broadcasting, the routing source node can only be a data sender. Figure 10 shows an example of how multicasting is used in a digital fronthaul end (DFE) NoC system.

Broadcasting packets are used to quickly transmit data or control information to the entire system for global reconstruction. The routing source transmits identical data to all nodes. When the broadcast indicator is set to high, other connections in the whole network are cleared. The source routing is set as the data sender, and all other nodes in the whole NoC are set as data receiver. The broadcasting packet has the highest priority. The broadcast packet structure is specified in Figure 7d.

### 4.3. Short Packet

As mentioned above, PCCNoC is designed to support relatively fixed task flows which usually have a large amount of data to transfer. In order to improve the ability of transmitting small amount of data, the proposed design provides a small packet transmission function. This kind of message is aimed at small data transmissions which can be packed in a single route packet. The packet can only be transferred from the routing source to the routing target through packet switching, and does not lock the circuit along the way. The packet structure is specified in Figure 7b.

### 4.4. Pipelines

Pipelines are divided into routing pipelines and data transmission pipelines.

1. Routing pipelines are used by the router to perform the routing function and other control information calculation. The routing packet consumes one clock at each node.
2. Data transmission pipelines are the pipelines used by router for the data transmission circuit. The data transmission circuit of each node can be set as a combination circuit or a pipeline register. Along the locked data path, most nodes are set as combination logic while few intermediate nodes are set to pipeline register according to the length of the whole path. The information about which nodes are set as pipeline registers is calculated by routing source.
3. If CRC is acquired, extra clock cycles are needed by the data sender and data receiver according to the port bandwidth. However, when parallel CRC is designed using Galois matrix, the extra cycle cost can be negligible.

**Figure 11:**
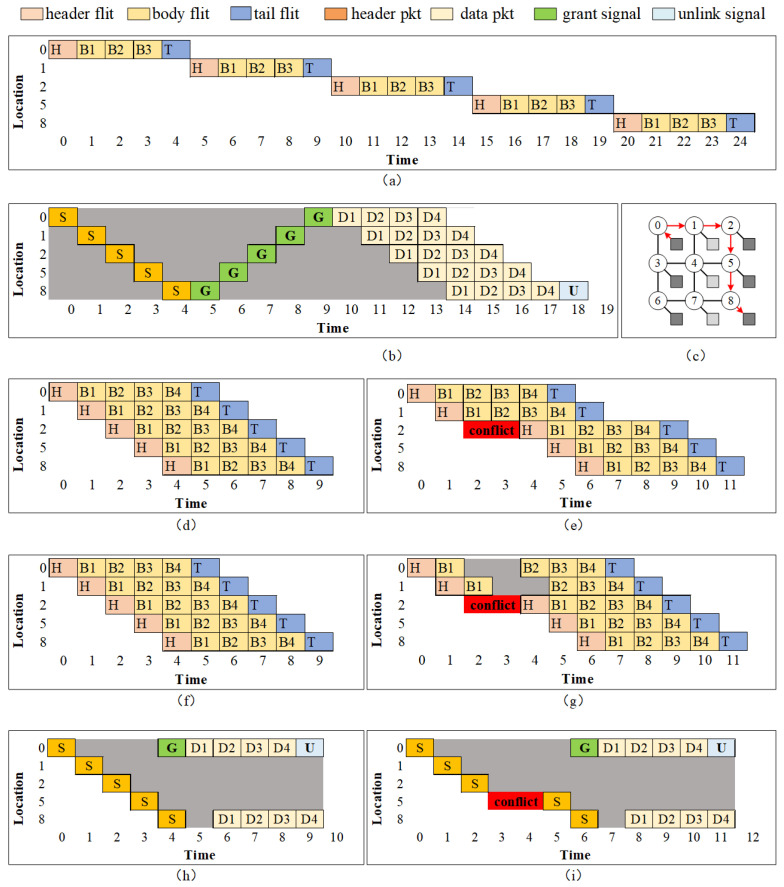
Pipelines for different switching method for a 3 × 3 mesh NoC using the adaptive X-Y routing method. The data transmission task is from node 0 to node 8, as shown in (**c**). The subfigures are: (**a**) PS cut through data flow; (**b**) Conventional CS data flow; (**d**) Virtual channel(VC) PS data flow; (**e**) VC PS data flow with conflict at location 2; (**f**) Wormhole(WH) PS data flow; (**g**) WH PS data flow with conflict at location 2; (**h**) Packet connected circuits (PCC) data flow; (**i**) PCC data flow with conflict at location 2. Because the datapath is short, the grant signal and unlink signal are purely combination logic.

Figure 11 depicts difference in pipelines between PS NoC and PCCNoC. PCCNOC establishes a transferring circuit at first, which take one time unit going through every node. After the circuit is connected, it takes only one time unit to transfer a packet from DS to DR. VC NoC and WH NoC transfer flits by pipeline, each flit is transferred immediately with the former flit. When conflicts happen, VC NoC continues to transfer flits to conflict-adjacent nodes while WH NoC stalls the transfer link until the conflict is resolved. In theory, the transferring time is the same for VC, WH, and PCCNOC. However, PCCNOC can accelerate data transfer after a circuit is established, as only one time unit is required for each packet and the transmission time is guaranteed. Each node only consumes one clock cycle per routing process, while VC PS NoCs and WH PS NoCs need extra cycles for buffering and data-ordering [8].

## 5. Implementation and Evaluation

System configuration time, latency, throughput, and area/power consumption are the main indicators used to measure NoC performance. For heterogeneous multi-core real-time system on chip, fast data transmission between different cores can improve system performance. In this section, we analyze and compare the performance of NoCs.

### 5.1. Latency and Throughput

First, a set of experiment is designed to compare the latency and throughput between PCCNoC and conventional PS NoCs. Noxim [40] simulator is used to model and simulate the traditional packet switching NOC. Noxim is a well-known cycle accurate NoC simulator to analyze performance of a NoC. In order to justify the performance of the proposed work, routers and network interfaces for PCCNOC have been implemented in Noxim. The PCCNoC is compared with a standard VC PS NoC model on Noxim. The detailed NoC configurations are presented in Table 2.

**Figure 12:**
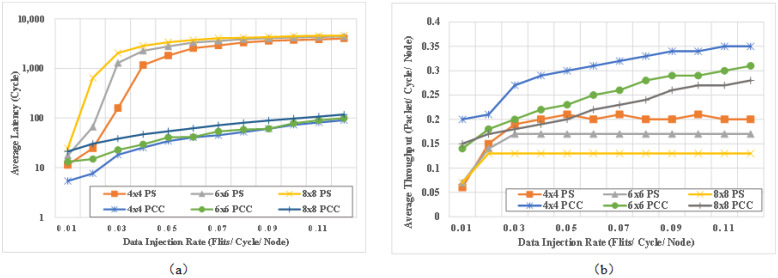
Comparison for (**a**) average latency and (**b**) average throughput for different NoC with different IRT.

Figure 12 shows the comparison of simulation results with different mesh size under different injection rates (IRT). Average latency refers to the average clock delay of messages arriving at the destination, and average network throughput refers to the the average packet number received by destination node at every clock.

From Figure 12, it can be seen that the average packet delay of our scheme for mesh networks with different sizes has been significantly decreased thanks to the extremely low delay of circuit switching after packet switching, which reduces the overall average packet delay. It can be observed from the figure that the average network throughput has been significantly improved, as the established circuit can achieve full bandwidth transmission.

In addition, it can be seen that performance saturation point of conventional PS NoC occurs when the injection rate is 0.08. By contrast, the average delay and network throughput of PCCNoC have good linear characteristics with variation of injection rate, and the saturation point is much higher. For injection rate 0.12, the throughput of PCCNoC improves 32%, 61%, 85% respectively for 4 × 4, 6 × 6 and 8 × 8 PS Noc and the latency decreases 92%, 95%, 95% respectively.

Transmission length is set as another experimental variable. Because the data port bandwidth is firm, we adjust the packet number for every transmission to achieve the transmission length variation effect. The size of every packet is 4 bytes and additional 7 bits are used for PCCNoC. The detailed NoC configurations are the same with Table 2 except that the packet number changes for every experiment and the injection rate keeps 0.01.

**Figure 13:**
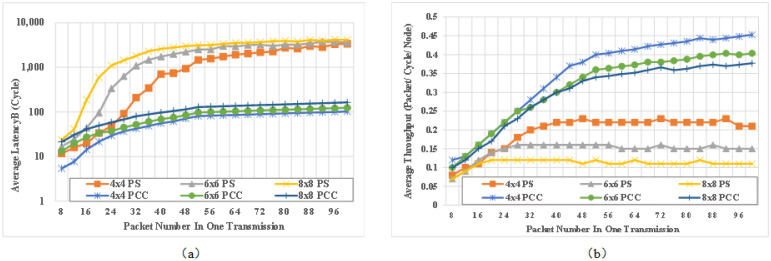
Comparison of (**a**) average latency and (**b**) average throughput for different NoC with different flit numbers.

Figure 13 shows the comparison of simulation results with different mesh sizes while the packet number varies. It can be seen that the average latency of the conventional packet switched NOC network increases with packet number increasing, and the network throughput is saturated after a slight improvement. The throughput saturation points are 24, 32, 40, and 56 packets in a transmission, respectively, for 4 × 4 PS NoC, 6 × 6 PS NoC, 8 × 8 PS NoC, and PCCNOC. As the packet number increases, the average throughput of PCCNoC keeps increasing and its average latency remains basically unchanged. The throughput of PCCNoC improves 115%, 169%, 242% and the latency decreases 96%, 97%, 97% respectively compared with 4 × 4, 6 × 6 and 8 × 8 PS Noc for transmission task with 100 packets (400 bytes). Because each packet for PS NoC needs to be routed, when there are more packets to transmit there is a higher risk of router congestion.

In PCCNoC, however, the data payload is transmitted on a locked circuit, and all packet-related drawbacks disappear. With the increase in Transmission length, the delay of PCCNoC tends to flatten out, while the network throughput increases greatly. Thanks to the fast, low-delay, and high-throughput circuit switching after the datapath is locked, PCCNoC is suitable for heterogeeous multi-core real-time SOC. If the interface bit width is set to 256 bits in real application scenarios, the improvement is even more obvious.

**Figure 14:**
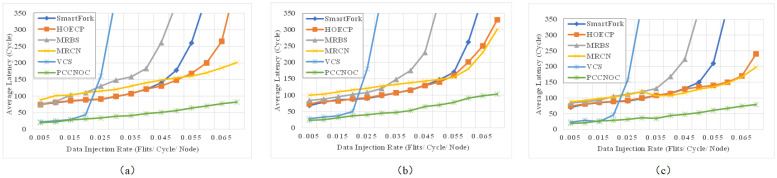
Average latency simulation results in the 8 × 8 mesh node under (**a**) uniform random traffic, (**b**) CTG-based traffic, (**c**) Rent’s rule traffic.

Simple traffic models such as uniform random are useful for NoC designers in acquiring insights by stressing the network with a regular predictable traffic pattern. However, they do not represent realistic traffic loads, which are necessary to assess NoCs [41]. Thus, utilizing traffic models that mimic realistic traffic behaviour, such as CTG-based and Rent’s rule, is significantly important. CTG-based traffic injects packets according to the traffic rate between each communication node in the task graphs generated by FTTG [42]. Rent’s rule uses communication probability matrix to send packets to each node uniformly, obtain synthesis flows, realize the communication locality [43]. Our experiments were designed to trace NoC behaviour under different traffic models with an 8 × 8 mesh NoC using Noxim. Figure 14 depicts the average latency according to the injection rate in an 8 × 8 mesh NoC. The performance of PCCNOC was examined with VCS [7], MRBS [9], MRCN [8], and SmartFork [14]. It can be seen that the saturation point of injection rate for PCCNoC is higher than all other related work, and the average latency is lower at injection rates from 0.005 to 0.065 for the all three test cases. For the injection rate at 0.04, the average latency reduction achieved by PCCNoC is 59%, 58%, 72%, 62%, and 93% in comparison to Smartfork, HOECP, MRBS, MRCN and VCS, respectively.

Next, we focus our attention on real benchmark traffic. Snipersim 6.1 [44] was used to trace real benchmark traffic, and the router and network interface of PCCNOC were implemented on Snipersim. We selected five PARSEC [45] benchmark applications (viz. vips, X264, fluidanimate, blackscholes, and dedup) due to their variability in offering both communication and computation-intensive workload. The performance of the proposed technique was examined against a baseline packet switching NoC (namely, PS) and two similar works: Dancyflyer [15], which supports selection of multipath output port under dynamic path selection policy, and VCS [7], which reserves a virtual path for routing packets in an adaptive mode for each communication flow. Both methods support in-order packet delivery. The simulation was executed on an 8 × 8 mesh NoC and the execution time was 100,000 cycles.

**Figure 15:**
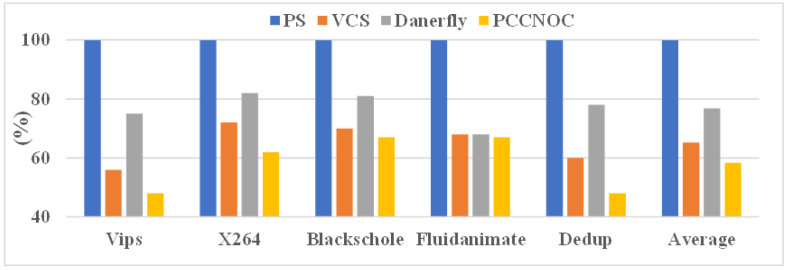
Throughput (in Mbps) normalized to PS on 8 × 8 mesh under PARSEC benchmark suite.

In the case of PARSEC benchmark application, the maximum gain in terms of average throughput achieved by three techniques is observed as 42%, 35%, and 23% for PCCNOC, VCS, and Dancerfly, respectively, compared with the baseline (see Figure 15).

### 5.2. Configuration Time

Using an 8 × 8 mesh NOC architecture and an injection rate of 0.15, we compared it with two typical packet-switched NOC networks, AEthereal [26], dAElite [25], and a hybrid NoC network CTN [34] with configuration time. It can be seen from Table 3 that the system configuration speed of our method is 125 times faster than AEtheral, 10 times faster than dAElite, and 3.8 times faster than CTN. The main reason for this time improvement is the advantage of the fast path used for feedback on exceptions and the use of multicasting.

### 5.3. Synthesis Result

The router was implemented by Verilog. Each router was synthesized with Synopsys Design Compiler using the TSMC 12 nm standard cell to extract the timing and area information. The clock frequency was 3 GHz. For the real application scenario, the bandwidth was set to 263 bits (256 bits data and 7 bits for control). The synthesis results are listed in Table 4. It is worth mentioning that the area is the logic circuit overhead excluding the overhead metal connection wire.

In order to compare different methods, we configured the interface bitwidth to 49 bits. Due to the different processes used in different studies, we refer to the method in [46] to normalize the silicon area to 65 nm for comparison. Our work is compared with two hybrid switching NoCs CTN [31] and VCS [7] and three packet switching NoCs MRBS [9], namely, MRCN [8] and SmartFork [14]. Our proposal resulted in a decrease in area of 5.6% and 56.5%, respectively, compared with CTN [31] and VCS [7]. This is because we used the same interface to handle packet switching and circuit switching, and only increased the additional bandwidth of the 7 control bits. Compared with PS NoC, the area used by our proposal is reduced by 88.4%, 88.1%, and 67.9% compared with MRBS [9], MRCN [8], and SmartFork [14], respectively. Compared with PS NoC, the proposed method saves the storage overhead of the re-order buffer and virtual channel. The results of different studies are listed in Table 5.

**Figure 16:**
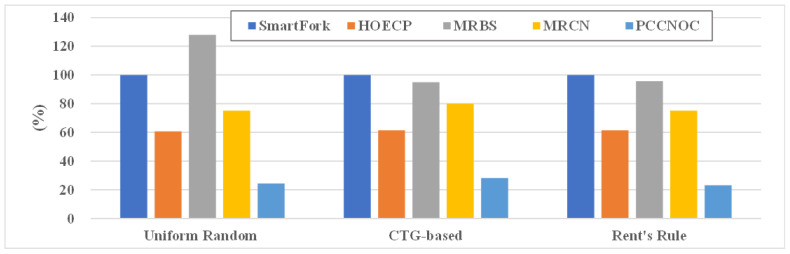
Comparison of energy-delay product with other work.

Additional simulation was conducted using the energy estimation function provided by Noxim. The power estimation function of Synopsys Design Compiler and the power model of the TSMC 12 nm Design Kit were applied in Noxim. The simulation was conducted using an 8 × 8 mesh NoC and an injection rate of 0.03 for variable control. Three different traffic models (uniform random, CTG-based, and Rent’s Rule) were used to drive the NoC. The simulation time was 100,000 cycles. Our proposed work uses the energy-delay product (EDP) to measure comprehensive performance, where the energy is the total energy consumption of the cores and the delay is the amount of time required to execute applications. SmartFork [14], HOECP [16], MRBS [9], and MRCN [8] were set as references, and the results were normalised to Smartfork. Figure 16 illustrates the EDP for different works under the three different traffic models. The average gain in EDP achieved by four techniques is observed as 38%, −6%, 23%, and 75% for HOECP, MRBS, MRCN, and PCCNoC compared with SmartFork [14]. These results show that our proposal has better comprehensive performance compared with related works.

## 6. Conclusions

The proposed design, called PCCNoC, provides an NOC architecture for a heterogeneous multi-core real-time system on a chip. PCCNoC inherits the packet switching advantages of flexible routing of packet switching and scalability in addition to the low latency of a circuit switch network. Furthermore, it is buffer-free, which is the key advantage of circuit switching. The data transferred on PCCNoC is based on a circuit, and the data are absolutely in order, meaning that the re-order induced latency area overhead is eliminated. This scheme can support multiple parallel transmissions at the same time. It is a hybrid NoC that uses packet transmission to establish a data path and lock this data path into a temporary circuit.

Compared with traditional packet switched networks, PCCNoC has 97% lower latency and 242% higher throughput, which is particularly suitable for complex heterogeneous multi-core real-time SoC, such as baseband systems in base stations. Compared with other related works, PCCNoC uses the same resource for packet switching and circuit switching, resulting in reduced area and power consumption. Moreover, it has multicasting and broadcasting functions, expanding the possible network-on-chip application scenarios, and in the future could be equipped with a CRC and timer to ensure QoS.

## Figures and Tables

**Table 1:** Detailed information for each part for router interface.

Port Name	Bit Width	Direction	Function
Data	N bits	DS to DR	Payload or packet.
Enable (EN)	1 bit	DS to DR	To indicates whether the transmission is valid
Unlink (UL)	1 bit	DS to DR	To unlock the constructed path
Overtime (OT)	1 bit	DR to DS	To notice the transaction has time violation
CRCe (CRC)	1 bit	DR to DS	To states the data transmission fails
Trans_grt (TG)	1 bit	DR to DS	To authorize data transaction of data sender
Trans_ref (TF)	1 bit	DR to DS	to refuse data transmission

**Table 2:** Detailed information for each part of the router interface.

NoC Model	Mesh: 4 × 4, 6 × 6, 8 × 8
Flit width	34 bits (2-bit flit type + 32-bit(4Byte) message data)
Packet length	12 flits (1 head flit + 14 body flits + 1 tail flit), total 48 byte message data.
Operation time	10,000 cycles
Traffic model	Uniform random
Route algorithm	adaptive-XY
Synthesis time	100,000 clock cycles

**Table 3:** Configuration time in cycles.

	AEthereal [26]		dAElite [25]		CTN [34]		PCCNoC	
	ideal	measured	ideal	measured	ideal	measured	ideal	measured
**Cycles**	246	1000	60	81	16	30	8	8
**Cost factor**	30.8×	125×	7.5×	10×	2×	3.8×	1×	1×

**Table 4:** PCCNoC router synthesis result.

	Bandwidth (bits)	Area (μm${}^{2}$)	Combinational Area (μm${}^{2}$)	Noncombinati-Onal Area (μm${}^{2}$)	Power (mW)
PCCNoC	263	3180.81	1406.78	1774.03	3.2

**Table 5:** Synthesis result comparison with other studies.

Research	Bandwidth (bits)	Technology (nm)	Frequency (GHz)	Area (μm${}^{2}$)	Normalized Area (μm${}^{2}$) ^1^	Power (mW)
CTN [31]	48	TSMC 65	2.8	44,157	44,157	29
VCS [7]	-	TSMC 45	2	46,000	95,975	17.1
MRBS [9]	34	SAED 32	0.5	87,753	362,066	11.8
MRCN [8]	34	SAED 32	-	85,616	353,249	8.5
SmartFork [14]	-	45	1	62,370	130,130	5.58
Our work	49	TSMC 12	3	1421	41,692	1.4

^1^ Normalized_ area = area $\;✕\;{\left(\frac{65\mathrm{nm}}{\mathrm{process}}\right)}^{2}$.

## Data Availability

The data that support the findings of this study are available on request from corresponding author.
